# Clinical Experiences in Private Practice, Etc.

**Published:** 1870-12

**Authors:** Z. C. McElroy

**Affiliations:** Zanesville, Ohio


ARTICLE IV.—*Clinical Experiences in Private Practice—the Modus Operandi and Therapeutic Uses of Veratrum Viride.* By Z. C. McELROY, M.D., Zanesville, Ohio.

The consulting physician referred to in my recent report of a clinical case,* read a paper to the Muskingum County (Ohio) Medical Society, at its August session, 1870, taking exception to the statement of fact in the history of the case, as published, during his connection with it, as well as the therapeutical conclusions drawn from it, which, by a resolution of the society, was ordered to be forwarded to the CHICAGO MEDICAL JOURNAL for publication, with the debate on the whole case. I voted for the resolution, and hope you will open your pages for their insertion; and, appended, this, my reply, for the reason that it is my impression it will do good.

The matter of the facts of the case, as published, may be safely referred to the parents of the child, whose statement, made at my request, is here annexed.† And, with that, the question whether my report of the case is correct or not may be safely dismissed. I, however, again declare, that the case as reported and published is substantially correct, as nearly so as it is by words possible to reproduce any case, with reference to critical judgment on its therapeutic management.

The part played by Veratrum Viride in the case is the real point at issue. Did its influence tend to carry the patient through the crisis? Or did it place him in greater jeopardy than he was before its administration? The purpose of this paper is to try to furnish solutions to these inquiries.

It is much to be regretted that the "impressions and opinions" of gentlemen in the scientific world, are not at all times held subject to correction by accomplished facts in reference to anything, even to the matter of the dose and mode of administering any therapeutic agent, let it be Veratrum Viride or anything else.

The fact that the patient was substantially convalescent in four

* Chicago Medical Journal, July, 1870.

† [The letter from the parents, containing the statement referred to by Dr. McElroy, is in our possession, but we deem its publication unnecessary. —EDS. JOURNAL.]

or five hours after the last dose of ten drops—thirty drops in all, from six to eight o'clock—cannot be explained away, or got over. He did get thirty drops in something near two hours—did vomit and purge involuntarily, and at the close of the "depressing effects" was substantially convalescent, and is here, alive, to-day, to show that thirty drops Fld. Ext. Ver. Viride, given, ten drops at a dose, at intervals of one hour, until he had taken three doses—thirty drops in all—did not finish him when he was already so near dead.

The author of the paper, and so many of the fellows of the society as participated in the debate, evidently consider, and indeed stated, Veratrum Viride to be a most virulent poison. And as no one of them defined what they understood by a poison, to get at their probable mental impressions reference must be had to lexicons.

Webster's definition is, "any agent capable of producing a morbid, noxious, or dangerous effect upon anything endowed with life." Dunglison's, "a generic name for all substances which, when introduced into the animal economy, either by cutaneous absorption, respiration, or the digestive canal, act in a noxious manner on the vital properties, or the texture of organs."

And these definitions may very properly be compressed into the conception of a gentleman whose wife had taken a little too much Belladonna for her asthma, for their ideas of her personal safety and comfort. Though prescribed by a physician, both husband and wife thought the exchange of her difficult breathing, for the apparent cutaneous heat, confused mental faculties, and nearly suspended vision, rather a poor bargain. And while he was soundly berating physicians for prescribing such "pisens," I asked him this question, "Mr. Palmatry, what do you understand by poison?" "Pisen, pisen, why, pisen is something that kills people."

This reply induced me, at that time (about a year since), to make further efforts to get at what was the prevailing mental conceptions in the popular and professional mind as to what was poison, and this reply of Mr. P. was found to reflect them very accurately in this city and county. And this debate, in connection with the definitions introduced here, from the lexicons in use by both people and the profession, shows, with tolerable accuracy, that the professional does not materially differ from the popular understanding as to "What is poison?"

However proper these conceptions may have been only a quarter of a century since, they are extra scientific in 1870, and not much to the credit of any gentleman, claiming to be scientific, who holds them.

But, working on this popular idea, or mental conception, of what is poison, how do the impressions and opinions of these gentlemen compare with the facts on record, "by authority" of the profession? Is Veratrum Viride a poison at all? Did it ever kill anybody? Did it ever produce noxious effects? or morbid? or dangerous?

Prof. Wood* says, "These signs of prostration are sometimes so great as to be alarming; although I have seen no account of fatal poisoning."

Prof. Stillé† says, "Alarming and apparently dangerous as these symptoms are, it does not appear that they have ever terminated in death. On the contrary, they have been dispelled with facility by merely suspending the use of the medicine, and administering diffusible stimulants."

Profs. Wood and Bache‡ are silent in regard to any dangerous or fatal effects. Nothing—not a word—is said about fatal poisoning. And if there is a recorded death from Veratrum Viride, it has never fallen under my observation.

Prof. H. C. Wood§ experimented with two alkaloids, obtained from the Veratrum Viride, Viridia and Veratroidæ, by hypodermic injections, on inferior animals. Viridia he found to depress directly the heart's action, and death by paralysis, but no post-mortem lesions of form, or vomiting, or purging before death. Veratroidæ he found, also, to depress the heart's action, death by asphyxia, violently emetic, sometimes cathartic, locally irritant, but no post-mortem lesions of form. With the resin of Veratrum on man, his experiments resulted, not in depressing the heart's action, but dyspeptic symptoms.

* Therapeutics and Pharmacology, II, 154. 1856.

† Therapeutics and Mat. Med., II, 343. 1860.

‡ U. S. Dispensatory, 1865.

§ Am. Jour. Med. Science, Jan., 1870.

So much for "authority;" and, in so far as authority deals with facts, none can yield more readily or gracefully to it than myself. But, in so far as "authority" deals in opinions and impressions, or inferences, it is known that for these there is in my mind little reverence. Opinions I can manufacture by the mile daily, and their value will be inversely to their length or number. Scientific conclusions and inductions are, with me, the result of mental and physical pains-taking labor, and needful delay, to obtain all possible accuracy.

My acquaintance with Veratrum Viride commenced about twenty years since, and for not quite half of that time I held opinions concerning it not materially different from those brought out by this discussion.

A dearly-loved female relative, who passed away ten years since, took, for several months before her death, a combination of Veratrum and Aconite, twice daily, to produce vomiting. They were combined, because results were obtained from both, which did not follow either separately, the reason for which will appear before I get through with the subject. At 9 o'clock in the morning she took the requisite dose, was sick by 10½ or 11; ready for her dinner soon after noon; in her carriage for a ride on all pleasant days, as free from discomforts as an incurable invalid could be; usually returning in time for supper. At 9 o'clock in the evening the same thing was repeated, falling asleep usually before midnight, and resting tranquilly till morning. This was found, by experience, to be the only way in which the inordinate working of an enlarged heart—a heart that had lost its normal form*—and, as a result, its normal function—and a plainly-to-be-seen aneurism of one of the external carotids—could be so controlled as to obtain the comfort she had from noon till evening, and from midnight till morning. All other means had been tried, and failed to give a moment's relief from the terrible — truly terrible restlessness which so soon overtook her after the effects of the Veratrum had passed off. She dreaded the vomiting all the time, would gladly have escaped it, but she dreaded the terrible restlessness more, and so, meekly and cheerfully submitted to the

* Chicago Medical Journal, January, 1870, p. 14

effects of the Veratrum; and it is proper to say, that under the vomiting dose, she did not obtain relief.

This opened my eyes, and enlarged my knowledge of Veratrum Viride. Had it been such an active and virulent poison as my fellows in the society insist on, this lady should have died from "symptoms of poisoning" months before she did. When her death did occur, it was peaceful, and, at the moment, unnoticed by an attentive and vigilant daughter, in her room, and sitting but a few feet from her at the time, as if she had only fallen asleep. She returned from her ride at 5½ in the afternoon, and before 6 o'clock she was dead.

A few months after this lady's death, a small child, perhaps a year old, daughter of Mr. Uriah Sharp (now a young woman, hale and hearty, and seen within a few days), helped herself to a vial containing about half an ounce of the tincture, and, unnoticed, swallowed its contents. As, in prescribing it in the family, I had explained to them that it was an active poison, and had it so labeled, they were greatly alarmed, and sent for me immediately. The mother had had presence of mind to compel the child to drink some mustard water, so that I found her vomiting. Placing the child's head very low, permitted it to vomit. It became very pale; pulse sunk down very low, perhaps below 40, though not counted at the time. At a proper moment, a little spirits and paregoric was administered, repeated as it was thrown up, until the vomiting ceased, and the pulse rose in frequency. Returning in a few hours, found the child, to my very great surprise, playing, as if nothing had happened.

I was considerably wiser in regard to Veratrum Viride in consequence of this accident.

Not long after this occurrence, and during the same year (1861), I had two patients in one house, both married females, under treatment for continued fevers. For one of them Veratrum had been prescribed, so that she would get eight drops in a teaspoonful of a mixture, once in six hours; and a Citrate of Potassa mixture, of which she was to get a tablespoonful every two hours. By mistake, the Veratrum mixture was given in tablespoonful doses every two hours from morning till evening. At my evening visit, she was found sick, very sick, at her stomach, had a ball in her throat which she thought would soon smother her, had vomited quite a number of times; pulse very slow, skin very cool, and, in professional technology, "greatly prostrated." The cause was soon ascertained, and a glass of brandy, with some paregoric, administered, with speedy relief. This patient, though considered much the worse of the two, recovered at once; but in the other case, which did not get Veratrum at all, the fever ran a course of three weeks.

I did not fail to learn something from this mistake, too. And to mistakes, sometimes my own, but more frequently others', I am indebted for much of the knowledge I now esteem valuable in my vocation. Early in my professional career, incidents began to undermine my confidence in the validity of what I had been taught in regard to therapeutics, as a medical pupil. They culminated in my setting about, a year or so after these occurrences, studying the problem, "How do medicines produce their effects?" the results of which were communicated to the profession in a monograph, near the close of 1868.* By this time, and in consequence of this labor, my knowledge of Veratrum had been considerably enlarged. Working away at the problem, the physical, physiological, and dynamic unities of organic life were reached toward the close of the year 1869, and first communicated to the profession in the January number of this JOURNAL for the present year (1870). But before this time, I had determined, by experiment, that the best results of Veratrum were realized from a single large dose, given to produce vomiting and the very "depressing effects" so much feared by the fellows of the society, and to caution others against a like dangerous use of the drug, was the purpose of the paper, debate, and its publication.

From this gradual accumulation of empirical personal experiences, extended over twenty years, my deliberate judgment now is, that a teaspoonful, or, for that matter, an ounce, given at a single dose, to produce these identical "depressing effects," so much deprecated by certain fellows, is, for certain conditions, accompanying high temperatures (105° to 108°), rapid circulation, great jactitation and restlessness, very much safer, and followed by infinitely better results, than when given in smaller doses,

* Chicago Med. Jour., March 1, 1869, p. 157.

simply with the view to controlling the heart's action. This condition of things is mostly due to retention of the results of tissue metamorphosis, for which I find no designation more accurate and expressive than dead matter, for the expulsion of which the very excessive motion is set up in the interest of waste, of which the jactitation and restlessness are mere incidents. And it seems to me that these better results are due to the elimination of this dead matter, or results of tissue decay. With such a view and purpose it was given to Albert Patterson, when the other measures instituted to the same end had failed, and it seems to me the results were satisfactory, and expectations founded on this purpose were fully realized.

The consultation, I own, did temporarily unsettle my purpose, from 8½ A. M. to 5 P. M., but it was then resumed, and never deviated from till its close; I had no occasion to regret it, and think I learned something from the episode.

Had Albert received brandy, and "diffusible stimulants," so called, it seems to me tolerably certain, that Mr. and Mrs. Patterson would have had a funeral from their residence within a couple of days, and I should not have met and spoken with him only yesterday (August 22, 1870).

Since Albert's case was treated, I have given it in half and teaspoonful doses a good many times, and with uniformly and altogether satisfactory results. The conclusion, then, is forced on me by these experiences, that this is one of the proper and legitimate uses of Veratrum Viride in practical therapeutics.

Science is cosmopolitan. Her temple has no side or back door, through which to smuggle a falsehood, so that it may pass for a truth or reality. If a teaspoonful of tincture or fluid extract of Veratrum Viride can be safely given in Zanesville, it will be equally safe to repeat it in Chicago, London, St. Petersburgh, or Melbourne, under like circumstances. But art is not so. Chicago may at this moment hold a human hand, or human head, which can do that which not another in the world can do so skillfully. And I aver that a teaspoonful of a good article of tincture or fluid extract Veratrum Viride can be given, even by inexperienced hands, to check excessive motion, and eliminate dead matter, under such circumstances as were presented in Albert's case, in Chicago, Zanesville, San Francisco, or any other place, and at any time, without fear of death from its "depressing effects," or "poisonous results," such as were pictured to the society during the debate.

All that any therapeutic, remedial, or hygienic agency, or measure, can, by any possibility, do, or any mode of force, mechanical, chemical, or physical, to any human body, sick or well, dead or alive, can be merged, nay, will go without violence, or straining, or distortion, into one of these three classes; and cannot be made to go into any other, aiming to be fundamental or ideal unities, viz.: 1st, Retarding motion in the interest of repair or waste, i.e., constructive or destructive metamorphosis; 2nd, Promoting, or accelerating motion in the interest of repair or waste; 3rd, Changing the molecular forms of structure, or tissue, or viscera. I repeat, that nothing on, above, or below the earth's surface, can do anything else than one of these three things, to a human or organized body, dead or alive.

Aside from my own experiences, those of Prof. H. C. Wood, so recently reported, and heretofore alluded to in this paper, demonstrate that neither Viridia, Veratroidæ, nor the resin of Veratrum, changes the molecular forms of structure, as small pox, scarlatina, etc.; and, therefore, as it only influences motion, or chemical changes in the molecular forms of structure, that influence, when accompanied or succeeded by elimination, by vomiting, or vomiting, purging and sweating, does not, or rather has not, up to this time, "killed" anybody, and will not be likely to, so long as it is followed by active elimination.

Prof. Wood thinks that in Viridia the profession has a remedy, by means of which, "all the peculiar sedative influence of Veratrum Viride can be obtained, without the horrible nausea and vomiting which always occasion so much discomfort, and, in some instances, endanger life." He does not say that it does anything more than "endanger life." And suppose that his expectations in regard to Viridia are realized, it will prove to be a much more dangerous agent than the tincture of the whole drug; and for the reason that the "horrible nausea and vomiting" are absent, which are the sure guarantees of safety to life. It must, then, be placed with aconite, gelseminum, and chloral—agents which retard motion without elimination, and, therefore, agents which can, and will, in large doses, destroy life. The popular, as well as professional, ideas of "virulent," or "deadly," or "active poisons," are all based on the velocity with which motion, or chemical changes are promoted, retarded, or arrested, or forms changed. The form-changing "poisons" are, virus of serpents, insects, etc.; combustion (burning); mineral acids, (concentrated); certain salts, as chloride of zinc; the concentrated alkalies, and their oxides, and included in this class are the physical forces, falls, mechanical violence—as by machinery; blows; bullets and bombs, etc., etc.; for the reason that, if they do not change forms of molecular structure, they rarely, or perhaps never, destroy life. And the measure of danger is the extent of the change of molecular forms of structure. Man and animals become old simply because the normal molecular forms of structure, as well as physical contour, or figure, are changed, or lost. And old age is not considered a "poison," simply because the changes of the molecular forms of structure are spread over a lifetime—come gradually, slowly, but none the less surely.

Other modes of force in chemical combinations of matter, influencing motion, or chemical changes, in both the interest of repair and decay, depend for being considered "poisons" solely on the velocity with which they influence motion.

Thus, prussic acid and the cyanide of potassium are considered among the "deadly" poisons, solely on account of the velocity, or rapidity, with which they retard, or arrest motion, or chemical changes in the molecular forms of structure. In this class are chloral and chloroform. Strychnia, on the other hand, is considered a "deadly" poison, solely on account of the rapidity with which motion, or chemical changes in the interest of waste, is promoted.

Happily, the greater number of remedial agents in use only influence motion in either, or both, the interests of repair and waste. A few change forms, as calomel, and from them people shrink, they cannot tell exactly why, but the reason, for calomel, is, changing molecular forms (salivation). Tartar emetic, Spanish flies, etc., change forms, and promote motion in the interest of waste, and are not popular remedies, and probably never will be, and are very likely to pass further out of use by the profession.

I think, therefore, that the results of empirical experience alone, which, by the by, is the court of final resort in all vexed or unsettled therapeutic questions, aside from the philosophy of its operation, demonstrates that Veratrum Viride simply retards motion in the interest of waste, and eliminates dead matter, but does not change molecular forms of structure, when given in doses sufficient to produce vomiting, or vomiting, purging and sweating; and that, when indicated for these purposes, a teaspoonful, or even larger single doses, is safer; though, for that matter, either is safe enough, and better results are realized than from the same quantity in smaller doses, extended over more time.

P. S. It may be proper to state, that when Veratrum is given in a large dose, for the first time in a family, I never leave the house until the vomiting ceases. When the vomiting comes on, chairs are placed under the posts of the foot of the bed, pillows removed, and a basin held to receive the discharges, giving tepid water through a glass tube, or, in its absence, a straw. I deem this due the patient; and though it looks distressing to bystanders, patients rarely complain about it after the sick stomach ceases. The relief quite compensates for the apparent distress.

